# The New Organization of Ethics Committees in Italy: What is the Future of Clinical Ethics?

**DOI:** 10.1007/s11673-024-10389-1

**Published:** 2025-02-13

**Authors:** Pietro Refolo, Costanza Raimondi, Dario Sacchini, Antonio Gioacchino Spagnolo

**Affiliations:** Research Center for Clinical Bioethics and Medical Humanities (CRiBCeMH), Università Cattolica del Sacro Cuore, Fondazione Policlinico Universitario “A. Gemelli” IRCCS, Rome, Italy

**Keywords:** Clinical ethics, Ethics committees, Research ethics committee, Clinical ethics committee, Territorial ethics committee, Local ethics committee, Ethics consultation service, Italy

## Abstract

In Italy, clinical ethics is not well institutionalized. On February 7, 2023, the Italian Ministry of Health published four long-awaited decrees regarding the reorganization of ethics committees. *Aim:* The aim of this article is twofold: firstly, we aim to briefly summarize the development of clinical ethics in Italy from a legislative point of view; secondly, we aim to examine how Italian regions are implementing the part of the new decrees on the organization of ethics committees that concerns clinical ethics. *Methods:* As for the first aim, we conducted a critical interpretive review (CIR). The search was restricted to the opinions offered by the Italian National Bioethics Committee (CNB) and to the major Italian legislative decrees on the topic. Regarding the second aim, we conducted an online search through Regional Official Bulletins of each Italian region. *Results:* Our analysis showed that despite the recommendations from the CNB to differentiate Research Ethics Committees (RECs) and Clinical Ethics Committees (CECs), over the years legislative attention has mainly focused on RECs and pharmacological matters. The new decrees allow regions to be flexible in organizing their activities. However, it emerged that only four regions (Veneto, Friuli-Venezia Giulia, Puglia, Emilia-Romagna) have split the roles, while all the other regions have entrusted both roles to a single committee. *Conclusion:* The risk for Italy is to take a step backward in the development of clinical ethics. Possible solutions could be either making Local Ethics Committees (CELs) mandatory or institutionalizing Ethics Consultation services (ECSs).

## Introduction

Clinical ethics can be defined as “a form of applied ethics practised in the hospital or health care setting and concerned with actual clinical choices” (Boyd et al. 1997, 40). Its main purpose is to “improve the quality of patient care by identifying, analysing, and attempting to resolve the ethical problems that arise in practice” (Siegler et al. 1990, 264).

Clinical ethics differs from research ethics, which governs the standards of conduct for scientific researchers. While research ethics is promoted through the so-called Research Ethics Committees (RECs),^1^ which are tasked with safeguarding the safety and welfare of human subjects involved in research studies, clinical ethics deals with issues arising from clinical practice. The two fundamental approaches that have been adopted for achieving the essential goal of clinical ethics are Clinical Ethics Committees (CECs) and Ethics Consultation Services (ECSs) (Singer et al. 1990). Clinical ethics requires different expertise, and it operates following different procedures from research ethics. Moreover, clinical ethics organs, when consulted, generally provide non-binding advisory opinions. For all these reasons, it seems beneficial and necessary not to assign two very different tasks to the same body.

Indeed, it is possible to trace the birth of clinical ethics committees in North America, a beginning that may overlap in terms of timing with that of research ethics because of a growing sensibility overall, but one that has its own specific milestones that characterize its different nature. The first case that made it clear that clinical ethics needed a specific space is the establishment of the Admissions and Policy Committee of the Seattle Artificial Kidney Center at the Swedish Hospital (Seattle, United States) in 1962. The role of this body, made up of seven lay people chosen as representatives of community ethical standards, was to decide which patients would get access to dialysis machines, since the centre did not have the capacity to provide the treatment to all those who needed it (Jonsen 2007). The members were tasked with making such crucial decision with no moral or ethical guidelines other than their own individual consciences, which is the reason why the committee was later called the “God Committee.” This episode made it clear for the first time in a very apparent way that medical practice needed a space where a “systematic, critical, reasoned evaluation and justification of right and wrong, good and evil in clinical practice” could take place (Sulmasy 2001). Other well-known cases that can be seen as milestones for the growth of clinical ethics and the establishment of CECs in the United States include Karen Ann Quinlan (1975), Baby Doe (1982), and the Cruzan case (1990) (Aulisio 2016).

Formal ECSs in hospitals are more recent. The first clinical description of the process and outcome of a formal ECS was not published until 1987 (Singer et al. 1990). In 1992, the Joint Commission on the Accreditation of Healthcare Organizations (JCAHO) passed a mandate requiring that all accredited hospitals find a mechanism to address ethically complex cases and concerns (Zivot 2020). After that mandate, the field has greatly developed.

CECs and ECSs were soon established in Europe as well. Although it cannot be denied that their birth was inspired by what was occurring in the United States, in Europe the connotation of such organizations was quite different. American CECs mainly emerged from the need to support doctors with technical (i.e., legal and ethical) knowledge that they were lacking and that was needed in order to address complex clinical cases. Moreover, their role was also to defend medical authority, which was being questioned more and more alongside the rise of the patients’ rights movement: one could say that they were bodies born *of* the institution (Furlan 2016). The same cannot be said about Europe, where medical authority was not facing such questioning: there, CECs were not seen as damage control bodies for the doctors, but rather as a space in which to think of the new challenges of medical practice. Their role was not so much a functional one in terms of technical knowledge, but rather a democratic one. One could say that European CECs were born not as bodies of the institution, but as bodies *within* the institution in the context of the healthcare system (Furlan 2016; De Panfilis, et al. 2019; Crico, et al. 2021).

The aim of this article is twofold. Firstly, we aim to briefly summarize the development of clinical ethics in Italy from a legislative point of view. Secondly, we aim to examine how Italian regions are implementing aspects of recent decrees on the organization of ethics committees that concern clinical ethics. These four long-awaited decrees regarding the full implementation of Regulation (EU) No 536/2014 on clinical trials of medicinal products for human use were mandated by the Ministry of Health in February 7, 2023. The primary scope of such documents is medical research, and therefore, they focus on the reorganization of RECs. However, they also address the issue of the reorganization of CECs. As a result, the future developments of clinical ethics in Italy are strictly linked to the implementation of such decrees.

## Materials and Methods

To trace the development of clinical ethics in Italy, we conducted a critical interpretive review (CIR), a method that has advantages over traditional systematic reviews when addressing ethical and legal questions (Dixon-Woods et al. 2006; McDougall 2015). A CIR differs from the traditional systematic review of literature in that it aims to capture the key ideas relevant to a particular issue, rather than assembling every article relevant to the research question. To begin with, AGS, who has a longstanding focus on the topic of clinical ethics committees, identified relevant papers that were already known to him. Then, we added documents retrieved from Google Scholar on the major Italian legislative decrees up to these days as well as the opinions offered by the Italian National Bioethics Committee (Comitato Nazionale per la Bioetica (CNB)) related to the organization of ethics committees. Finally, we read the literature with a view to identifying the central ideas about how to manage clinical ethics in healthcare systems.

Regarding the implementation of the new decrees, we manually conducted an online search through Regional Official Bulletins (Bollettino Ufficiale Regionale, BUR) of each Italian region.

We collected the material, and the results were used to perform the analysis. An early draft of the article was prepared, becoming the starting point for further discussion among us. Finally, we integrated the different components of the analysis. Looking at the whole picture, we reorganized the results into three thematic areas: legislative development of clinical ethics in Italy, new decrees on organization of ethics committees in Italy, and implementation of the new decrees.

## Results

### Legislative Development of Clinical Ethics in Italy

In the 1980s, following the advice given in the first revision of the *Declaration of Helsinki* by the World Medical Association (WMA) (1975) and in response to other pushes for the normalization of systems for the oversight of clinical ethics, several bodies for the review and regulation of clinical trials started to develop throughout Italy, with no regulation or clear acknowledgement, differing greatly in composition and procedures.

In 1990, the CNB was established by a decree signed by the President of the Council of Ministers. It was born as—and still is—a consultive body for the Council of Ministers, and the tasks given to it were to develop a summary of the programs, objectives, and results of research in the field of life sciences and human health; to express opinions and give guidance to legislators on how to face the challenges posed by scientific progress and medical practice with regards to the protection of fundamental human rights and dignity; to promote the drafting of codes of conduct for professionals in the different fields involved; to encourage and promote correct and adequate information so the general public is informed on sensitive topics. Its birth stands as a clear example of what was argued earlier: the democratic role of clinical ethics has always been more prevalent than its functional role, which, as we will show in the present section, still struggles to find its way.

In 1992, two years after its constitution, the CNB released an opinion (Comitato Nazionale per la Bioetica 1992) on RECs, emphasizing the need for regulation. The document pointed to the growing complexity of healthcare services, naming, among others, assisted reproduction, genetic therapies, experimentation on humans as well as animals, resource allocation, availability of different healthcare treatments, the need to balance patient autonomy, and living wills and the issues that they bring to the doctor–patient relationship. The CNB expressed a concern, though in a very brief and incomplete way, that has matured over the course of the years—a concern that a single body would be insufficient to fulfil all the tasks required. The proposal at the time was to have two types of ethics committee: one that would operate at a regional level in order to serve and give recommendations to the regional authorities in their healthcare administration and management (resource allocation, experimentation, healthcare assistance planning, quality checks); the other type would operate at a local level, giving recommendations on research and clinical practice issues that emerge with difficulties requiring resolution.

A few months later, in 1992, the Italian government issued the first decree (*Disposizioni sulle documentazioni tecniche da presentare a corredo delle domande di autorizzazione all’immissione in commercio di specialità medicinali per uso umano, in attuazione della direttiva (CEE) n. 507/91*) in which they speak explicitly of “ethics committees.” Translating the European directive *Good Clinical Practice for Trials on Medicinal Products in the European Community*, the decree required that all research protocols comply with standards of good clinical practice and that any clinical trial be approved by a REC before starting. However, it should be noted that there was no mention of dealing with issues coming from clinical practice, limiting ethics committee activities to the realm of pharmacology (Viafora 1999).

Other decrees, (*Recepimento delle linee guida dell’UE di buona pratica clinica per l’esecuzione delle sperimentazioni cliniche dei medicinali* and *Linee guida di riferimento per l’istituzione e il funzionamento dei comitati etici*), issued in 1997 and 1998, further defined, respectively, the structure of the new committees and their relationship to the institutions they served, but for the most part they continued to be concerned only with clinical trials. Mention of clinical practice was only done in very incidental way, such as in the 1998 decree where it was written that such committees should deal with healthcare assistance, “where applicable.”

In the same time period, the CNB issued another opinion (Comitato Nazionale per la Bioetica 1997) questioning the feasibility of having a single type of ethics committee fulfil two functions, something that had never been explicitly recommended but nonetheless was overwhelmingly the case: little attention was given to clinical practice because no role was officially required for it. However, the opinion was a short four-page document, and the issue was posed, again, as a question rather than a recommendation for change.

A few years later, in 2001, the CNB changed their opinion. In a new document (Comitato Nazionale per la Bioetica 2001), they reflected again on other controversies regarding ethics committees, underlining a number of concerns such as the risk of only dealing with bureaucracy and the difficulty in complying with all its tasks. The opinion analysed the differences between ethics committees’ role as review boards for clinical trials (i.e., RECs) and the other roles that could and should be carried out. A paragraph was dedicated to “ethics consultation in clinical practice,” stating that it is without doubt one of the “most delicate and important” functions of ethics committee. It highlighted that while RECs’ role in clinical trials approval has been defined by many international and national documents, the role of ethics consultation has not. It granted that the presence of ethics committees can occur in different forms, such as retrospective or prospective discussion of a case or elaboration of recommendation and guidelines for common situations. However, the consultation offered by a single clinical ethics consultant was not taken into consideration. The opinion concluded that two separate committees, one for clinical trials and one for clinical practice, should be established and called for a legislative, not ministerial, intervention.

Two years later, in 2003, a legislative decree was indeed issued, but it concerned the implementation of good clinical practice in clinical trials on medicine. The legislator once again did not acknowledge the importance of the topic of clinical ethics.

Ten years after the request made by the CNB, in 2012, a legislative decree (*Disposizioni urgenti per promuovere lo sviluppo del Paese mediante un più alto livello di tutela della salute*)was mandated, which called for a reduction and equal distribution of RECs throughout the territory and stated their responsibilities should include the monitoring of the use of drugs and medical devices, as well as the practice of surgical and clinical procedures (Minacori et al. 2015). Once again, it was recognized that such evaluation must be carried out by a formal body, and there were signs of openness in broadening the task of RECs.

Such openness towards the issues coming from clinical practice was reinforced the following year with a decree (*Criteri per la composizione e il funzionamento dei comitati etici*) that assigned RECs advisory and educational roles on ethical questions connected to scientific and healthcare assistance activities.

Thus, RECs were tasked to stick to both roles, and the obvious consequence, as the CNB recognized in a 2017 opinion (Comitato Nazionale per la Bioetica 2017), is that the second function was not carried out. The 2013 decree had in fact reduced substantially the number of RECs in Italy, and such reduction was intensified with EU Regulation 536/2014.

Law n. 3 of January 11, 2018, by the Italian Parliament (*Delega al Governo in materia di sperimentazione clinica di medicinali nonchè disposizioni per il riordino delle professioni sanitarie e per la dirigenza sanitaria del Ministero della salute*) asked for the identification of a maximum of forty local ethics committees; however, the ministerial decree to enact this did not occur within sixty days, as requested within the law itself. This enactment occurred through the long-awaited decrees mandated on February 7, 2023.

In the 2017 opinion (Comitato Nazionale per la Bioetica 2017) on CECs, the CNB underlined their conviction that two bodies with different tasks were needed, as they had already affirmed in the 2001 opinion. Here, they articulated another aspect of the need for diversification: while clinical trials evaluation must follow strict protocols, clinical cases must be always considered in their unique singularity. For this reason, the CNB encouraged the legislator to establish them formally but still allowed a certain degree of flexibility in order that they may function more adequately for their task. For this reason, too, the wish was for them to be locally based and not too fragmented: each committee should know its own territory and reality, because of more direct contact with users and audience. In addition, the CNB recommended that CECs be independent from the institution or authority that hosts them and discussed its composition: it stated that a fixed number of members should be included in the committee with the possibility of adding other experts when needed. The members that should always be present were deemed to be a doctor, a bioethicist, a nurse, a lawyer, an expert on health risk management, a patients’ representative, and an epidemiologist. Those who could be called when needed were, according to the opinion, a chaplain, a social worker, a psychologist, and a cultural mediator, among others. Within this discussion about the members to be included, the CNB opened up a question that would be developed in a consequent CNB opinion (Comitato Nazionale per la Bioetica 2021): who is the bioethicist? This is an important question that is still being discussed to this day, both in terms of competences and in terms of formalized training. The document also discussed the task that would be assigned to such committee: to discuss and analyse all ethical dilemmas that come from the healthcare practice and assistance, whether they be related to the therapeutical choices to be made or the vulnerability of the subjects involved (such as minors, incompetent patients, elderly, and others). CECs would also be asked to carry out and supervise training initiatives for healthcare professionals and raise public awareness through other initiatives. They might be asked to carry out clinical consultation, to mediate when needed, and to provide support in the drafting of informed consent.

### New Decrees on Organization of Ethics Committees in Italy

As mentioned above, Law n. 3 of January 11, 2018, asked for a ministerial decree to mandate the identification of a maximum of forty local ethics committees within sixty days, but for years the controversy about the identification of such committees went on without any resolution.

On February 7, 2023, four decrees^2^ of the Ministry of Health regarding the reorganization of ethics committees were published in the government gazette. Their main aim is the full implementation of Regulation (EU) No 536/2014 on clinical trials on medicinal products for human use.

Two of the four decrees deal with the reorganization, the technical-organizational aspects, and the simplification of the ethics committees network, thereby completing a long process aimed at simplifying trial procedures, improving timing, and ultimately increasing Italy’s competitiveness in the research field. The other two, more technical, decrees are concerned, respectively, with the definition of a standard fee for clinical trials evaluation and the regulation of the transitional phase (Hogan Lovells 2023).

According to the new organization, which has removed the previous terminology, ethics committees are modelled on a three-level structure.

On the first level, there are three National Ethics Committees (CENs), whose competence is exclusively on advanced medicinal therapeutical products (ATMPs), paediatric clinical trials, and trials of public entities.

The second level comprises forty Territorial Ethics Committees (CETs). The Ministry of Health identified these committees, and subsequently, the regions will appoint them to such a role through regional decrees. CETs primarily handle pharmacological matters, such as trials on drugs (in collaboration with the Italian Medicines Agency, AIFA), clinical investigations on medical devices, and pharmacological observational studies. Additionally, CETs may also address non-pharmacological matters related to healthcare practice and assistance.

The third level is optional and consists of Local Ethics Committees (CELs), i.e. the further 90 CECs that currently exist and are not included in the forty that will be appointed to be CETs. They branch out to healthcare facilities based on a benchmark distribution of at least one of every million inhabitants. The regions can choose whether to keep the CELs active. When active, CELs will handle activities that do not fall within the exclusive competence of CENs or CETs, i.e., pharmacological matters (Hogan Lovells 2023); when CELs are not active, CETs will handle both roles.

Both CETs and CELs must be appointed by the regions and remain independent from the institutions or authorities that host them.

Regarding the composition of both types of committees, the decrees clarify that they must include three experts of experimentation, a local general practitioner, a paediatrician, a biostatistician, a pharmacologist, a hospital pharmacist, an expert in legal matters, an expert in insurance matters, a coroner, an expert on bioethics, a representative of the health professions with an interest in the trial, a representative of patients’ associations, an expert on medical devices, a clinical engineer or a medical physicist, an expert on nutrition, and an expert of genetics. For specific evaluations, both CETs and CELs may involve external experts recruited through public calls. Table 1 summarizes the main documents relevant to the development of ethical committees in Italy in chronological order.

**Table 1:** Timeline of the development of Italian ethics committees

Year	Type of Document	Institution	Main statement	Explicit reference to clinical ethics issues
1980s	No regulations			
1990	Decree	CNB	Establishment of CNB	Yes
1992	Opinion	CNB	Encourages two types of ethics committee: regional and local	Yes
1992	Decree	Ministry of Health	Compliance of research protocols with standards of good clinical practice	No
1997	Decree	Ministry of Health	Committees’ structure and organization	No
1997	Opinion	CNB	Questions the feasibility of having one kind of EC fulfill two functions	Yes
1998	Decree	Ministry of Health	Relationship of RECs with the institutions	No
2001	Opinion	CNB	Encourages establishment of two separate committees, one for clinical trials and one for clinical practice	Yes
2003	Decree	Ministry of Health	Implementation of good clinical practice in clinical trials	No
2012	Decree	Ministry of Health	Reduction and equal distribution of RECs	Yes
2013	Decree	Ministry of Health	Committees’ composition	Yes
2017	Opinion	CNB	Urges the establishment of CECs and outlines their characteristics	Yes
2018	Law	Italian Parlament	Identification of 40 local ethics committees	Yes
2021	Opinion	CNB	Definition and role of the bioethicist	Yes
2023	Decree	Ministry of Health	Reorganization of the ECs network	Yes
2023	Decree	Ministry of Health	Fee for clinical trials	No
2023	Decree	Ministry of Health	Regulation of the transitional phase	No

### Implementation of the New Decrees

Our analysis, which covered all 20 Italian regions,^3^ revealed that fifteen of them (Valle d’Aosta, Liguria, Lombardia, Piemonte, Toscana, Umbria, Marche, Lazio, Abruzzo, Molise, Campania, Basilicata, Calabria, Sardegna, Sicilia) chose not to keep their CELs active (see appendix 1). As a result, those CETs that have been selected by the Ministry of Health for each region will handle both pharmacological and non-pharmacological matters. More specifically, three regions (Lazio, Molise, and Sardegna) explicitly specified in their respective decrees that CETs have competence over activities related to both clinical trials and clinical practice, while the remaining twelve regions did mention the ministerial decrees but failed to explicitly refer to tasks linked to healthcare practice and assistance.

In this scenario, our analysis also revealed three exceptions: Veneto, Friuli-Venezia Giulia, Puglia, and Emilia-Romagna.

Veneto chose to keep active all CELs appointed by a previous regional decree,^4^ which had established that every Local Health Authority (ASL)^5^ must have its own committee supporting clinical practice. Similarly, Friuli-Venezia Giulia chose to keep all CELs appointed in 2016, referred to as “ethical nuclei,”^6^ tasked with supporting clinical practice for each ASL; and Puglia chose to keep active two CELs established in 2016 and 2017, both located in the north of the region. Finally, Emilia-Romagna chose to keep active one CEL appointed in 2020.

## Discussion

The premise of any consideration is that in Italy, there are no institutionalized ECSs. There are some well-established experiences, such as the Clinical Ethics Consultation Service at the “Agostino Gemelli” University Hospital Foundation IRCCS in Rome (Spagnolo et al. 2019) and the “Domus Salutis” Clinic in Brescia (Picozzi et al. 2016). Additionally, there are some educational programmes (Nicoli et al. 2017) and one scientific association called the “Interdisciplinary Group of Clinical Bioethics and Ethical Consultation” (Gruppo Interdisciplinare di Bioetica clinica e Consulenza etica in ambito sanitario (GIBCE)) (https://eticaclinica.wordpress.com/) (Zonza and Refolo 2014), which has been active since 2010, aiming to promote the culture of clinical ethics consultation. It could be said that overall, there is an increasing awareness of the importance of ECSs (Leuter et al. 2018); nevertheless, ECSs have never been institutionalized from a legislative point of view.

Our analysis showed that despite the CNB’s recommendations to differentiate RECs and CECs over the years, the attention of the legislators has mainly focused on pharmacological matters. The new decrees allowed regions to be flexible in organizing their activities. However, from the analysis, it emerged that only four regions have split the roles, while all the other regions have entrusted both roles to a single committee (CET).

It is worth noting the case of Puglia, where although two CELs have been kept active, both are located in the northern part of the region. Puglia is about 400 km long, and such CELs will be dealing with issues concerning healthcare facilities that are quite distant. With this in mind, can those two committees truly be considered “local”?

Given the current implementation of the decrees, it is inevitable that the CETs, also considering the significant workload they will have to face, will have limited time to discuss ethical issues coming from clinical practice. For the sake of context, one should know that the abovementioned Clinical Ethics Consultation Service of the Agostino Gemelli University Hospital Foundation IRCCS elaborated 176 clinical ethics consultations during the period 2016–2019, with a growing trend over the years (Spagnolo et al. 2019). The discussion of clinical cases is a time-consuming and complex activity that should be conducted at the patient’s bedside. Will the CETs be able to handle it, given the vast demand they are asked to face?

Furthermore, considering the absence of institutionalized ECSs in Italy and the fact that almost all regions have entrusted all tasks to CETs, healthcare facilities will lack dedicated spaces to address issues locally and individually.

To add complexity to such scenario, a recent ruling by the Italian Constitutional Court (Petrini 2019) assigns to ethics committees—pending a law on end-of-life issues—the task of verifying the legitimacy of requests for assisted suicide by patients according to four specific criteria. Are committees tasked with evaluating the ethics of experiments the most appropriate place to discuss such delicate practices?

Another point to highlight is that the recent decrees did not differentiate the composition of CELs from that of CETs. For example, both types of committees require the presence of three experts in experimentation. Is it appropriate? Do ethical issues related to clinical practice require the same expertise as those related to experimentation?

Lastly, the decrees contain a lot of information, details, and rules on how to carry out the evaluation of experimental protocols, while there is no indication regarding the evaluation of issues arising from clinical practice. This is undoubtedly due to the decrees being primarily concerned with clinical experimentation. However, since issue coming from healthcare assistance have been mentioned, perhaps it was necessary to provide additional information about them.

Overall, it seems to us that the new decrees on ethics committees and their implementation may significantly reduce the spaces for clinical ethics in Italy, potentially transforming it into an activity disconnected from local contexts and from the patient’s bedside. This seems to be the opposite to the trends in Europe where clinical ethics consultation services have been established in most of the transition countries of Central and Eastern Europe (Orzechowski et al. 2020).

## Conclusion

Our analysis showed that despite the CNB’s recommendations to differentiate RECs and CECs, the implementation of the new decrees on organization of ethics committee is leading to entrusting all tasks to a single committee. By doing so, the risk for Italy is to take a step backward in the development of clinical ethics.

Possible solutions could be either to make CELs mandatory or to institutionalize ECSs. In its 2017 opinion, the CNB expressed a preference for an organization based on committees (CECs). It goes beyond the scope of this article to evaluate whether the committee-based approach is better than the service-based one. We believe that relying solely on CECs may not be sufficient, as effective clinical ethics should be carried out at the patient’s bedside.

## Data Availability

All data generated or analysed during this study are included in this published article.

## Appendix 1. List of Decrees Issued by Italian Regions for the Implementation of New Decrees on ECs

Valle d’Aosta: Regional Decree June 5 2023, no. 627. Istituzione del Comitato Etico Territoriale della Regione Autonoma Valle d’Aosta.

Liguria: Regional Decree May 5 2023, no. 478. Costituzione del Comitato Etico Territoriale – Liguria.

Lombardy: Regional Decree April 28, no. 2023. Comitati etici territoriali- Fase transitoria dal 7 giugno 2023 al 31 dicembre 2023.

Piedmont: Regional Decree March 21, no. 24–6629. Attuazione dei Decreti del Ministro della Salute del 26, 27 e 30 gennaio 2023, inerenti all’Individuazione, alla composizione ed al funzionamento dei Comitati Etici territoriali (CET) ed alla determinazione della tariffa unica per le sperimentazioni cliniche. Revoca della D.G.R. n. 78–4807 del 04.12.2006, della D.G.R. n. 2–5737 del 23.04.2007 e della D.G.R. n. 25- 6008 del 25.06.2013.

Emilia Romagna: Regional Decree June 5 2023, no. 923. Costituzione Comitati Etici Territoriali (Cet) Della Regione Emilia-Romagna; Decree 13 February 2024, no. 194. Costituzione del Comitato Regionale per l'Etica nella Clinica.

Tuscany: Regional Decree July 6 2023, no. 120. Nomina dei Comitati Etici Territoriali della Regione Toscana.

Umbria: Regional Decree April 26 2023, no. 435.

Marche: Regional Decree May 15 2023, no. 627. Istituzione del Comitato Etico Territoriale (CET) con sede presso l’Azienda Ospedaliero Universitaria (AOU) delle Marche.

Lazio: Regional Decree April 28 2023, no. G05811. Riorganizzazione dei Comitati Etici Territoriali (CET) operanti nella Regione Lazio ai sensi del Dlg.s 3/2018.

Abruzzo: Regional Decree April 6 2023, no. 206. Armonizzazione delle disposizioni concernenti il CER della Regione Abruzzo ai Decreti Ministeriali sui Comitati Etici Pubblicati sulla Gazzetta Ufficiale del 7 febbraio 2023 Modifica Dgr 325/2021 Definizione Tariffe di Sottomissione degli Studi.

Molise: Regional Decree April 21 2023, no. 149. Legge 11 gennaio 2018, N. 3 e Decreto del Ministro della Salute del 26 gennaio 2023 – Riorganizzazione dei Comitati Etici Regionali per la sperimentazione clinica dei medicinali.

Campania: Regional Decree April 27 2023, no. 224. Riorganizzazione dei Comitati Etici Territoriali (CET) della Regione Campania. Modifica Dgr N. 597/2021.

Basilicata: Regional Decree February 2 2023, no. 202300089. DGR n. 755 dell’11/11/2022 e n.817 del 1. dicembre 2022.—Approvazione definitiva della composizione del nuovo Comitato Etico Unico Regionale (CEUR) di Basilicata a seguito del parere del IV Commissione Consiliare Permanente.

Calabria: Regional Decree June 7 2023, no. 7927. Approvazione graduatorie di merito e nomina componenti comitato Etico territoriale (CET) “Comitato etico Regione Calabria” e Formazione di un elenco di esperti ai sensi del decreto del 30 gennaio 2023 del Ministero della Salute.

Sardinia: Regional Decree April 20 2023, no. 15/9. Linee guida per l’Istituzione del Comitato Etico Territoriale della Regione Sardegna ai sensi della legge 11 gennaio 2018, n. 3—Comitato Etico Sardegna.

Siciliy: Regional Decree 3 March 2023, no. 18790.

Veneto: Regional Decree 29 March 2023, no. 330. Legge n. 3/2018 in materia di sperimentazione clinica e successivi provvedimenti attuativi: riorganizzazione della rete regionale dei comitati etici per la sperimentazione clinica.

Friuli-Venezia Giulia: Regional Decree 26 May 2023, no. 816. 3/2018. Delega al Governo in materia di sperimentazione clinica di medicinali nonché disposizioni per il riordino delle professioni sanitarie e per la dirigenza sanitaria del Ministero della Salute. Ricostituzione CEUR.

Apulia: Regional Decree 22 May 2023, no. 712. Riorganizzazione dei Comitati Etici della Regione Puglia in attuazione dei Decreti del Ministro della Salute del 26, 27 e 30 gennaio 2023 – Definizione modalità di nomina dei componenti dei Comitati Etici Territoriali (CET) e Locali (CEL) in fase di prima applicazione della norma nazionale e misure per assicurare il passaggio di funzioni tra i Comitati Etici.

Emilia Romagna. Regional Decree 13 February 2024, no. 194. Costituzione del Comitato Regionale per l'Etica nella Clinica.

## Footnotes

1 RECs, known as Institutional Review Boards (IRB) in the United States, are dedicated to evaluating research protocols and safeguarding the rights and welfare of human subjects enrolled in biomedical research.

2 Ministry of Health, Italy. Ministerial Decree January 26, 2023. Identification of forty territorial ethics committees; Ministry of Health, Italy. Ministerial Decree January 27, 2023. Regulation of the transitional phase pursuant to Article 2, paragraph 15, of Law No. 3 of January 11, 2018, concerning the evaluation activities and the modalities of interaction between the Coordination Center, the territorial ethics committees, the national ethics committees, and the Italian Medicines Agency; Ministry of Health, Italy. Ministerial Decree January 30, 2023. Determination of the single fee for clinical trials, the attendance allowance, and the reimbursement of expenses for participation in meetings of the National Coordination Center for Territorial Ethics Committees for Clinical Trials on Medicinal Products for Human Use and Medical Devices, as well as for Territorial Ethics Committees and National Ethics Committees; Ministry of Health, Italy. Ministerial Decree January 30, 2023. Definition of the criteria for the composition and functioning of territorial ethics committees.

3 Trentino-Alto Adige is the only region that, to date, has not implemented the decrees. For this reason, it has been excluded from our analysis.

4 Regional Decree June 17 2014. Regulation of the Ethics Committees Network: reorganization of provisions relating to the Regional Bioethics Committee and the Ethics Committees for Clinical Practice. Amendment of Regional Council Resolutions No. 4049 of December 22, 2004, No. 2870 of October 4, 2005, No. 4155 of December 18, 2007, No. 2520 of August 4, 2009, No. 519 of March 2, 2010, and No. 1081 of July 26, 2011.

5 In Italy, a Local Health Authority (ASL) is a public organization, whose primary scope is to deliver healthcare services to a specific territorial area (typically at the provincial level). The primary responsibility of an ASL is to perform the tasks of the National Health Service (NHS), as well as other duties established by law within the specified territorial area.

6 Regional Decree January 22 2016, no. 73. Establishment of the Regional Ethics Committee for Clinical Trials.
